# The Role of Stigma Management in HIV Treatment Adherence

**DOI:** 10.3390/ijerph16245003

**Published:** 2019-12-09

**Authors:** Lance Rintamaki, Kami Kosenko, Timothy Hogan, Allison M. Scott, Christopher Dobmeier, Erik Tingue, David Peek

**Affiliations:** 1 Department of Communication, University at Buffalo, Buffalo, NY 14260, USA; cmdobmei@buffalo.edu (C.D.); erikting@buffalo.edu (E.T.); 2 Department of Communication, North Carolina State University, Raleigh, NC 27695, USA; kamikosenko@gmail.com; 3 Center for Healthcare Organization & Implementation Research, United States Department of Veterans Affairs, Bedford, MA 01730, USA; Timothy.Hogan@va.gov; 4 Department of Population and Data Sciences, University of Texas, Southwestern, Dallas, TX 75390, USA; 5 Department of Communication, University of Kentucky, Lexington, KY 40506, USA; allison.scott@uky.edu; 6 Department of Medicine, Pen Bay Medical Center, Rockport, ME 04856, USA; davidmpeek@gmail.com

**Keywords:** HIV, stigma, disclosure, adherence

## Abstract

Social stigma is linked to improper HIV treatment adherence, but how stigma impairs adherence outcomes is poorly understood. This study included 93 people living with HIV in the United States who participated in focus groups or one-on-one interviews regarding how stigma might affect medication management. Latent content analysis and constant comparative techniques of participant responses that were produced three thematic groupings that described how participants (a) orient to HIV stigma, (b) manage HIV stigma in ways that directly impair treatment adherence, and (c) manage HIV stigma in ways that may indirectly impair adherence. These findings illustrate the need to understand how patients orient to HIV stigma when prescribing medications and the complications that are inherent to such assessments. In addition, these findings provide a simple framework for organizing the different ways in which stigma management strategies may disrupt treatment adherence. Conceptually, these findings also offer a paradigm shift to extent theories on disclosure and concealment, in which only disclosure has been cast as an active process. These findings demonstrate how concealment is far from a passive default, often requiring enormous effort. Ultimately, these findings may guide intervention programs that help to entirely eliminate HIV by promoting optimized counseling and subsequent treatment adherence.

## 1. Introduction

HIV remains one of the great pandemics of our time, affecting well over a million people in the United States and tens of millions world-wide [1]. Fortunately, the advent of highly active anti-retroviral therapies (HAART) in the mid-1990s radically improved the lives of those that were affected by the virus, providing a means of suppressing the virus from replicating within the body [2,3]. These therapeutic advancements turned the tide in the fight against this disease, reducing both morbidity and mortality rates resulting from HIV infection. In many instances, HAART medications brought those that were diagnosed with AIDS (a clinical diagnosis signifying a severely compromised immune system) back from the brink of death, providing hope for a chance at relatively healthy, productive lives [4]. As a result of these breakthroughs, clinicians now treat the disease as a manageable, chronic condition, rather than a terminal illness [5]. What is more, the precise application of HAART regimens can suppress the virus to such a degree that transmitting proves extremely unlikely [6,7], offering a real hope of gradually wiping out the disease entirely.

However, despite their many benefits, HAART therapies are no panacea. In addition to a plethora of undesirable side effects, these medications must be taken on a precise, routine basis in order to work properly. Even minor deviations from prescribed regimens provide the virus with replication opportunities, which promote the genesis of treatment-resistant strains of HIV and subsequent clinical failure [8]. Not only do these resistant strains impair the treatment of their immediate hosts, but they can also be later transmitted to others, leaving those newly infected with fewer therapeutic options available for combating the disease.

Given the urgency of proper adherence, patient education and counseling on the importance of adherence is paramount [9,10]; however, effective counseling interventions require facile knowledge of the factors that facilitate or impede proper adherence [11]. As such, considerable research has gone into identifying and understanding a range of such variables, including regimen characteristics (e.g., regimen complexity, scheduling, and side effects), social and psychological factors (e.g., mental health, identity, attitude towards disease and treatment, and social support), institutional resources (e.g., access to healthcare and availability of services), and personal attributes (e.g., gender, ethnicity, age, socioeconomic status, substance abuse) (for review, see [12,13]). Identifying which of these factors best predict adherence proves to be crucial in the development of educational interventions that are designed to bolster adherence outcomes, which must provide a clear focus while addressing multiple adherence barriers at once [10].

Few illnesses have been as stigmatized around the globe as HIV [14], concerns for which appear to be one of the most significant factors associated with (im)proper treatment adherence [15]. Patients’ concerns regarding being stigmatized for having HIV have been linked to impaired treatment adherence in studies around the world [16,17,18,19,20], across age groups [21,22], across socioeconomic status [23,24], and across racial and ethnic groups [25,26]. In addition to being ubiquitous across socio-demographic contexts, the magnitude of stigma’s relationship to adherence outcomes is profound. For instance, one study revealed that those with a high concern for HIV stigma were 3.3 times more likely to report nonadherence than were those with low concerns for HIV stigma [27]. These findings are striking, as they were revealed after controlling for a plethora of other variables that were previously shown to affect treatment adherence outcomes. In fact, when the researchers controlled for the aforementioned demographics commonly-associated with poor adherence, concern for stigma proved to be the only significant predictor of adherence outcomes.

The means by which stigma impairs adherence outcomes is poorly understood, although a number of studies demonstrate the connection between stigma and poor adherence. Much of the research linking these variables has been quantitative in nature [28], often providing correlational data or descriptive measures that fail to reveal the behavioral pathways between stigma and (non)adherence. Qualitative research, to date, has provided more insight on this connection, but most simply present participant accounts of concern over stigma being a barrier to proper adherence [16,29]. One notable exception is provided by Golin and colleagues [30], in which participants explicitly articulated experiencing a dilemma when having to take medications while in the public eye. Subsequent studies mirror these findings, speaking to the fear that patients experience over what others may think when seeing them taking their HIV medications [31,32]. In particular, these studies suggest that people might choose to forego their medications as a form of HIV-status concealment.

The disclosure dilemmas illustrated in these studies underscore the need for relationships between stigma and adherence to be further explicated from a communication perspective. Unsurprisingly, both disclosure management and HIV-related communication issues have long been the focus of academic interest among public health and health communication researchers. Conceptually, research in these areas displays an array of reasons why people disclose their HIV-status, placing special emphasis on concerns for stigma [33,34]. In addition, communication scholars and allied researchers have collaborated to develop theoretical frameworks that depict the processes that people undertake when deciding if and when they will disclose this information [35,36,37,38,39]. Specifically, these theories present a complex system of pros and cons to disclosure measured in unison with consideration for contextual and relational factors that drive decisions to disclose HIV status. More recently, health communication researchers have begun to examine the effects of specific forms of HIV status disclosure, including those that may be more or less likely to prompt stigmatizing responses [40].

Taken together, this work illustrates the rationale people living with HIV employ when deciding if, when, and how to disclose their HIV-status to others; however, the way in which disclosure is privileged in this research might distract from the significance and strategies of HIV status concealment. This might be due, in part, to viewing disclosure (not concealment) as an active process and, therefore, the one on which we might focus when considering privacy management from a communicative perspective. However, research on social performance has a long history of considering all of what we do when presenting ourselves to others [41,42], which readily encompasses how we hide information about ourselves. Concealment is not a passive, default position in social performance; rather, it can conceivably take many forms and require enormous effort.

Where focusing on strategies people employ to conceal their HIV status broadens the conceptual lens of disclosure research, doing so in the specific context of HIV medication adherence holds significant practical and clinical value. In particular, given that previous research demonstrates that HIV status concealment is connected to HIV treatment adherence, understanding what and how concealment strategies affect adherence is of urgent importance. A multi-phase study was conducted to address this lacuna, which focused on barriers to and facilitators of HIV treatment adherence and HIV stigma management. Participants’ in-depth depictions of how stigma management intersects with HIV treatment adherence reveal the complexity stigma’s role in affecting treatment adherence, both through direct and indirect pathways.

## 2. Materials and Methods

As part of a larger project on HIV self-management, three sets of interview data were collected regarding how social stigma affects HIV treatment adherence, following institutional IRB approval and approval from each of the respective healthcare sties at which data were collected. The three sets of interview data included (a) phase 1, which involved 14 men living with HIV who participated in four focus groups, (b) phase 2, which included 69 men living with HIV who participated in in-depth, one-on-one interviews, and (c) phase three, which included 10 women living with HIV who participated in in-depth, one-on-one interviews. For phases 1 and 2, a convenience sample of 83 participants was recruited through the infectious disease units at three Veterans Administration (VA) hospitals in a large, Midwestern city in the United States. As no women were recruited during phases 1 and 2 (no women were actively seeking care for HIV through these facilities at the time of recruitment), phase 3 was later employed to recruit 10 women living with HIV through three HIV service organizations that were located in a mid-sized, Northeastern city in USA. Recruitment for all three phases entailed passive solicitation via handouts that were distributed on-site by infectious disease staff (e.g., posted on common area information boards). Participants interested in taking part in these studies contacted the lead author, who then scheduled them for either a focus group or one-on-one interview, depending on the phase of the study. The participants received $25 in remuneration for their contributions. Content saturation was determined by the lead author (who conducted nearly all of the interviews), who coupled the redundancy of responses offered by participants with the participant total, which far exceeds quantitative expectations for saturation [43,44,45].

The study utilized grounded theory techniques during data collection, which involved reformulating and refining research questions as the study progressed to pursue promising lines of inquiry [43]. Specifically, this process employed an initial set of questions pertaining to stigma and treatment adherence during the focus groups, data that were then used to refine and expand questions used during the in-depth interviews. In addition to facilitating this grounded-theory technique, this two-tiered process was utilized to produce a data set that combined the synergy of focus groups with the depth of responses afforded by one-on-one interviews [46,47].

In phase 1, the lead author led four focus groups through a semi-structured interview regarding participants’ experiences managing their medications, including key barriers and facilitators to adherence, such as how concerns for HIV stigma could affect adherence directly or by disrupting key health-maintenance behaviors (for specific questions employed, see Appendix A). Probe and follow-up questions were included, when appropriate, to clarify issues and validate the interviewer’s interpretations of responses. These focus groups lasted 120 min each. Based upon the findings from these focus groups, refinements and additions to the questions were made to explore HIV stigma management and its subsequent role in treatment adherence. These questions were then employed during phases 2 and 3, which involved two of the authors conducting in-depth, one-on-one interviews with new participants, which lasted between 45 and 230 min (mean = 66 min). The digital recordings of the focus groups and interviews were transcribed verbatim and distributed to each member of their respective coding teams.

The focus group and one-on-one interview transcripts were both analyzed using latent content and constant comparative techniques [48,49]. As previously indicated, this included two stages of analysis, with the first stage following the focus group interviews and the second stage following the one-on-one interviews. In phase 1 analysis, two authors first reviewed the focus group data to identify the focal themes among the participants’ responses, so as to identify and refine interview topics that required clarification or further exploration during the one-on-one interviews. In phase two, which began after the one-on-one interviews were transcribed, all the members of the research team coded the transcripts for focal themes. Team members analyzed the data in their entirety, independently producing categorical schema for describing participant accounts, and then convened to compare and compile their findings. Through discussion and consensus regarding their respective compilations, the authors produced a categorical system for describing how participants (a) orient to HIV stigma, (b) manage HIV stigma in ways that directly impair treatment adherence, and (c) manage HIV stigma in ways that may indirectly impair adherence. As a content validity member check [50], the lead author presented coded findings to a focus group of 15 study participants to confirm the interpretations and proper presentation of results, all of whom agreed with the findings, as presented.

## 3. Results

Of the 93 participants in the study, 83 (89.2%) were men. In terms of racial composition, the majority (51.6%) identified as African American, whereas 34.4% identified as Caucasian, 13% as Latino, and a single individual identified as Asian. In addition, the majority of participants (68.8%) identified as heterosexual. The participants ranged from 23 to 71 years of age (mean age of 42.2 years), with a length of diagnosis ranging from less than one year to 21 years. In terms of clinical health, 83% of participants knew their viral loads, which ranged from undetectable (i.e., less than 50 virons per mm^3^) to 1400 (mean = 412), meaning that those who were knowledgeable about these clinical variables were largely in good health at the time of the study, despite the fact that the majority of participants (56%) had received an AIDS diagnosis at some point in time (see Table 1 for more).

The coding process produced three thematic groupings, describing how participants (a) orient to HIV stigma, (b) manage HIV stigma in ways that directly impair treatment adherence, and (c) manage HIV stigma in ways that may indirectly impair adherence. Following the table denoting the main and sub categories of these findings (Table 2), each category is discussed below, while using direct quotes from study participants as illustration. The authors selected their quotes based on their capacity to illustrate the category that they represent. Pseudonyms are used throughout the presentation of these quotes in place of participants’ legitimate names.

### 3.1. Orientation to HIV Stigma

Participants depicted a range of concern for HIV stigma, including its prevalence and potential severity for them. This, in turn, gave rise to varying degrees of concern over HIV stigma.

#### 3.1.1. Perceived Prevalence of HIV Stigma

The orientation to HIV stigma involved how participants described the significance of HIV stigma in society, the potential threat posed by others’ responses to disclosure of HIV status, and the degree of concern or anxiety they felt about others learning their own status. With regard to societal attitudes towards HIV, there was consensus among the participants that HIV stigma was widespread. George explained, “it’s everywhere. You can’t get away from it. People are scared and don’t understand it. We hate what we don’t know”. Similarly, Beth stated, “I’ve moved three times before [coming] here. I saw it everywhere. It doesn’t surprise me no more. I expect it. And don’t trust no body, because you don’t know how they really are. If you tell the wrong person, you’re in real trouble”. Even those who had yet to be targets of HIV stigma were mindful and expected to encounter stigma at some point. Stigma was, in a sense, an ax waiting to fall. As Don explained, “No, I’ve never had anything like that [referring to experiences with HIV stigma]. I know it could happen though. It could come from anywhere”. When asked to elaborate, Don explained, “Look, when you have this disease you have to know that every person you talk to might not like you because you have it. It’s just the way people are. Someday it probably will [happen]”. In this regard, participants mimicked a social variant of Wagner & Curran’s [51] Worried Well, having never been stigmatized, but fully expecting to be at some unknown point in the future.

#### 3.1.2. Perceived Threat of HIV Stigma

When asked to explain what forms HIV stigma might take, the participants listed repercussions for personal relationships, degraded social networks, loss of employment, loss of housing, and even physical harm, including death. As Jordan explained,

I’ve been around people before I had it and they talk about it like it’s a nasty person’s disease and they want to stay away from anyone with it. They don’t want to touch nothing that they’ve handled. It’s just one of those things that people are afraid of. I’ve lost friends. I have cousins who pretend I’m dead.

Christopher received warnings about disclosing his status. As he tells it, “I was talking to someone at the bar. I said I was HIV positive, and he says, “don’t’ say that!” He says, “I am too, but don’t say that at the bar! The bar will throw you out”. Similarly, Martin had first-hand experience witnessing others be stigmatized for their HIV, including in the workplace. As he explained,

Ninety-nine percent of people don’t know I have HIV. If they did, I wouldn’t have a job. I’ve handled claims where people were terminated from their jobs because they had AIDS. You’ve seen the movie Philadelphia. That’s nothing new. They won’t use that reason for terminating someone. I don’t care how good of an investigator I am, if my company found out I had it, I would be gone. They wouldn’t use that for an excuse, they’d come up with something else. That’s why I can’t get insurance- or try to get insurance- through my employer, because I’d have to tell them what I’ve got. That would get back to my employers, so I’d be out the insurance because I’d also be out a job. It’s not contagious, as you well know, but the stigma is still there. It’s still out there. It’s not as bad as it was ten years ago, but it’s still there.

Finally, Mike talked about how people become targets once others learn of their HIV status, and his perception that people with HIV might be left without recourse when they encounter such treatment. As he explained,

Once they find out that you’ve got the virus, “oh no!” They just shut off. A lot of people still don’t understand it. I had my kids taken from me and the reason why I don’t have them back now is because I’m HIV positive. But the court ain’t gonna say that. They’re going to beat around the bush, but point blank that’s what it is, because my stepdaughter is telling my kids that I’m cutting myself and I’m feeding my blood to them some kind of way. And these are kids. And they they’re not aware that the virus dies when air hits it. So they don’t know the difference, ya know? But as long as we got grown people brain washing other people about this virus, we’ll never live a normal life.

#### 3.1.3. Concern for HIV Stigma

Although this range of threats was considerable in scope and gravity, the participants’ reported concerns over HIV stigma illustrated a range of concerns along a continuum between low concern over HIV stigma and high concern for HIV stigma. Those whose responses were depicted low concern stated they cared little, if at all, about other people’s feelings, attitudes, or opinions regarding HIV. Comments, such as, “I don’t give a good goddam what anyone else thinks”, “I’m a grown man”, “I couldn’t care less”, and “they don’t pay my bills”, were examples of participants who claimed to be unconcerned with other’s dispositions toward them and their HIV status.

When participants who expressed low/no concern over HIV stigma were asked if HIV stigma in any way affected their medication adherence (or how they took their HIV medications), the responses were a resounding “no”. As Carl explained, “It doesn’t make any difference. I don’t take my medications because of what society thinks”. Similarly, Tom stated, “I don’t worry about what people think. I’m taking it for my benefit, not theirs, so whatever they think, I really don’t care”. This applied in both public and in private venues, as explained by Curtis:

It does not matter if somebody is there or not. This happened last week where I had a couple people over and somebody had a female friend there and I just went in the fridge, took out my pills, popped’em in my mouth, popped things back in place, and put it back in the fridge. That’s that. I don’t care. This is for me. My health. My life.

Of course, there were those who expressed some concern in varying ways without being consumed by it. For instance, Joey explained, “you gotta be aware of this stuff, and I do worry some, but I can’t let it run my life”. Similarly, Kristin said, “I try not to let it bother me, even though it still does. I know there are some people out there who will think bad of me. It is what it is. Still, life goes on and so do I”.

Despite the indifference to HIV stigma expressed by some, evidence appeared suggesting that certain participants may have presented a false bravado when claiming to have low concern for HIV stigma. Specifically, there were inconsistencies between early claims about being indifferent to others’ attitudes and later statements by the same individuals that showed clear concern over what others would think or do if they discovered the participant’s HIV status. For instance, when first asked about his concern over other people stigmatizing him, Miguel stated, “I really didn’t care, cause I’m gonna be me regardless of what you feel about me, what people would think about me. All the people on the street, it really doesn’t make no difference what they think”. In contrast, Miguel later explained how he hides his HIV status from others for fear of their reactions: “I really don’t put myself in the predicament where people could judge me. Who are we to judge? I don’t need that”. In another instance, John first stated, ““I don’t give a s--t what other people think. If they’ve got a problem with it, f--k’em”. Later, he admitted, “I don’t let them know [at work] because there’s no reason for them to [know]. I don’t know what their personal feelings are about it and I don’t want to take a chance”.

Given the range of reported problems concerning HIV stigma, it is unsurprising that other participants reported having great concern for others learning about their HIV status and what would follow if they did. As Maggie explained, “People can really hurt you. That’s why I don’t tell anybody [about my status]”. In a similar vein, Glenn stated:

I have friends who’ve got diabetes, and they get looked at like they’re pariahs. If they get looked at like pariahs because of diabetes, how do you think they’re going to look at me? So no way do I tell people.

### 3.2. Stigma Management and Adherence

When asked if concerns over HIV stigma (how others would treat you) affected how they took their medications, participants described a host of strategies for managing HIV stigma through concealment that caused them to forego or delay their medications. In many illness contexts, having a partial medication dosage in one’s system is better than none at all; however, in the case of HIV, this can be catastrophic. When HIV medications are delayed, even by small margins, the incomplete medication dosage creates a partial suppression of the virus, which promotes the production of treatment-resistant strains of HIV [8]. As such, delaying medications in this context can be as problematic as missing doses altogether.

In addition to stigma management strategies that directly impaired proper treatment adherence, participants also described strategies that indirectly impaired adherence. Largely, these were through concealment strategies that impaired the receipt of adherence support, or that led them to forgo information that would aid in treatment adherence. The efforts that were taken to conceal HIV status that directly or indirectly impair treatment adherence are detailed below, as are the contexts in which these strategies were employed.

#### 3.2.1. Direct Impairment of Adherence

Out of concern for stigma, participants reported going to great lengths to prevent others from discovering their HIV medications. Like many of the participants, Michonne was on a treatment regimen that required her to take medication while at work. Concerned over unwelcome questions that her pills would raise, Michonne developed a simple strategy for concealing them: “I just wait for a chance to go off to the bathroom and take my meds in the stall with me”. Hiding medications was a common theme in these data, with Carol offering up a similar dilemma when with friends or family: “Like when I was on vacation, that was especially hard when my family would go someplace and then I’d hide my medications, then I’d just sneak off and take them sometime”. Even a night on the town must be orchestrated around medications, as was the case with Daryl: “If we go to a restaurant, I don’t want people to see me taking my meds. If I have to take my pills and I can’t get away, I’ll probably just wait until later”. When asked, Daryl admitted, “Sometimes I forget after”. In this case, an intended delay led to forgoing a dosage altogether, all for the sake of concealing HIV medications from the public eye.

Of course, public venues were not the only contexts in which the participants took pains to conceal their medications from others. Rick explained how such efforts may even be required at home: “If I know I’m having people over, I’ll be able to plan for it. But if people just come over and we’re hangin’ out, I don’t want to be like, ‘sorry, I gotta go take a bunch of pills’. Or if I can’t take’em with nobody around, I might take’em later”. Of course, as evidenced by Daryl above, intentions to delay a dosage can readily result in skipping it altogether.

Eugene illustrated a particularly worrisome scenario. Like several of the study participants, Eugene rented a room at his house in order to help make ends meet. Concerned over his roommate’s possible reaction to learning of his HIV status, Eugene went to considerable lengths to ensure that his roommate never saw his HIV medications: “I don’t want him finding them, seeing them, so I keep them [HIV medications] in my car. I keep them in the trunk”. When asked how he takes these meds, Eugene explained, “I try to take’em when he’s not around or if I’m alone”. When asked, he admitted, “yes, sometimes I don’t take them if I can’t do it in private”.

Aside from the efforts that were taken to conceal his medication and HIV status from others, Eugene’s case illustrates another potential impairment to proper adherence: Improper medication storage. HIV medications cannot be warmed past a certain point, lest their active ingredients begin to break down. In fact, several HIV medications must normally be kept refrigerated [52]. In Eugene’s case, a car trunk under a Midwestern summer sun is a far cry from a refrigerator. In addition, freezing these medications can be equally problematic. In short, Eugene’s example illustrates how efforts to conceal HIV medications and HIV status can compound delayed or skipped doses through the inadvertent destruction of the medications, themselves. What is more, a patient unaware of what they have done is unlikely to discover a problem until their next visit with an immunologist, which could be months away. In the interim, they are likely taking degraded or wholly inactivated medications, promoting viral resistance, and losing ground on vital clinical outcomes, such as viral load and CD4 count.

#### 3.2.2. Indirect Impairment of Adherence

Although the participants described a host of ways in which their efforts to conceal their medications directly led to delayed or missed doses, disrupting their overall adherence and increasing the chances of experiencing clinical failure, they also recounted ways in which their management of HIV stigma indirectly impaired treatment adherence. This included problems in receipt of social support from others, including from those who were or would support their medication regimens. In addition, participants described ways in which they avoided treatment information altogether, even when they felt that they could benefit from it, all as a way of concealing their HIV status. Each of these strategies are detailed below.

**Social Support Management.** Social support is a vital means of promoting medication adherence, including in the context of HIV [53,54,55]. Support providers can remind patients to take their medications, help them to gain access to their medications, and help manage the side effects incurred by these drugs. However, many of these study’s participants forsook adherence support by entirely concealing their HIV status. As Jody explained simply, “I keep it a secret, so nobody helps me with it [medication adherence]”.

Recent studies have reported similar findings, with efforts to conceal one’s HIV status preventing people from accessing social support for its management [31]. These findings turn up an unusual spin on this phenomenon; however, where some participants employed a stigma management strategy that allowed them to conceal their HIV status while still fully accessing adherence support from friends, family, and coworkers. As Hershel explained, “I tell people I got a heart thing. I tell them that’s what I need to take medications for”. Tyreese used an identical strategy, only saying he has a stomach syndrome. “I use that to explain why I get sick some times and have be off work. People ask me how I’m doing all the time, especially when I’ve been off work. They even get after me to take my meds. They just don’t know what they’re actually for”. By using this small deception, the participants are bypassing the potential stigma of HIV while garnering the emotional support (e.g., expressed concern) and instrumental support (e.g., reminders to take medications) that promote proper treatment adherence.

Another novel finding involved how concerns regarding stigma can plague those who have shared their status with close contacts, such as family and friends, triggering management strategies that can undermine the support that these contacts might provide. Bob demonstrated how good intentions by loved ones can create situations that leave a person living with HIV feeling exposed and vulnerable. As he explained,

My wife, she looks out for me. I know she’s trying to help, but I don’t want her asking me if I took my pills when I’m in front of other people. I know it’s cause she cares. I just don’t want her asking me in front of them.

When asked what he does in these circumstances, Bob said, “I give her the look. She knows not to ask anymore, but sometimes she still does. I say, ‘*yes I did*’, and move on”. When asked why this was a problem, Bob explained, “I don’t want people asking me what I’m taking pills for”. Bob also explained how these benevolent gestures can leave him in a foul mood, even when he knows his wife is trying to help, which can cause conflicts between them. “She won’t talk to me afterwards sometimes. We’re both mad”. In this case, when supportive others are chastised or hurt by responses to their efforts, one might question whether the days in which that support continues are numbered.

**Information Management.** Concerns regarding stigma and the concealment strategies for managing it extended to how some participants manage information about HIV and their HIV medications. Specifically, several participants explained how fears over other people discerning their HIV status led them to alter how they sought and stored information regarding their medications. Shane was one such participant, who explained how he managed the medication information he received from his physicians and pharmacists when prescribed HIV medications: “I leave it [pamphlets and info about medications]. I throw it out before I leave. If I bring it home and forget it, someone’s just gonna find it and read it”. Similarly, Abraham explained a process by which he switched the bottles in which he kept his HIV medications, placing them in a Tylenol bottle before throwing out the prescription bottle. In both cases, the participants were trying to mitigate the chances of others discerning what their medications were for. In so doing, they destroyed the often-complicated instructions that came with these medications. When a patient does this and relies on memory to follow their regimen, they must remember the exact number of pills that they are taking per day, how many times per day they take them, whether they need to take them with food or on an empty stomach, how the medication must be stored, and the like. Clearly, the potential for error is great, with the discovery of such errors likely months away at follow-up visits, by which time viral resistance or worse may have occurred.

**Information Avoidance.** Finally, the concealment strategies for managing HIV stigma led participants to limit their access to or altogether avoid information sources regarding HIV and their HIV medications. The overriding concern across these narratives was the potential for others to discover these documents or otherwise track their searches for such information. For instance, Merle was one of several participants who lacked a personal computer. As such, he relied on public computers, such as those at the VA hospital or local libraries for accessing health-related information. Despite having access to such resources, Merle explained, “I don’t use [the internet] for that. You don’t know who’s gonna look over your shoulder and see. Or if they can track you. You know, Big Brother. They’re watching”. Although Merle made this statement while laughing at the end, it underscores a bona fide fear that is echoed by several participants over the potential for others to subsequently discern one’s HIV status. However, by avoiding these informational resources, they limit their own access to information that may prove beneficial to properly managing their medications (e.g., how to manage side effects, alternative regimens, and the like).

## 4. Discussion

Despite the progress made at educating the larger public about HIV and reducing HIV-related stigma on a large-scale [56,57,58], concern for HIV stigma appears to play a considerable role in how people adhere to their HIV medications. Although some participants in the current study described little concern for stigma, other participants feared stigmatizing responses to HIV disclosures and concealed their HIV status, as a result. These concealment strategies, which involve altering treatment schedules, skipping doses, and taking pills in private, help to preserve secrecy of one’s HIV status, but also directly threaten treatment adherence and efficacy.

Participants also described stigma management strategies that indirectly disrupt adherence. These included avoiding treatment-related information and adherence-promoting social support from others to avoid accidental exposure of one’s HIV status. Although several studies indicate a relationship between social support and treatment adherence [53], little is known regarding how social support might mediate stigma concerns or how stigma concerns may prevent access of social support [27]. This study provides evidence that stigma concerns impact social support and information seeking for individuals with HIV; however, quantitative research is needed to clarify the directionality, magnitude, and frequency of these effects. It is possible that avoiding information and support exacerbates stigma concern and its effects on treatment adherence. Future work regarding stigma concern should attend to these possible pathways to and effects on HIV treatment adherence, as well as treatment adherence in other stigmatized health contexts (e.g., opioid addiction).

Although most of the stigma management strategies that are presented appear to disrupt proper treatment adherence, one appears to facilitate it. Specifically, this involved a form of deception, in which one passes off HIV medications as necessary treatments for another illness altogether. This clever strategy garnered sympathy and encouragement for those managing their medications, potentially under the very noses of people who might otherwise be openly hostile toward someone with HIV. Future research is needed to further assess the utility, effectiveness, and drawbacks of this and similar deception strategies.

In addition to revealing novel stigma management strategies, these findings call into question issues in the broader literature pertaining to how researchers and clinicians conceptualize how people orient to HIV stigma. Specifically, these findings suggest that it may be a continuum of concern, as opposed to binary in nature. Many participants in the current study expressed high concern for HIV stigma; yet, there were also those who showed varying degrees of concern, from low to middling. Even those who were comfortable and informed about the virus were mindful that others may lack such insight, which made them cautious to varying degrees. The broader literature speaks to the heightened sensitivity with which people belonging to stigmatized groups may orient to those around them, remaining constantly on-guard against signals of dislike or rejection [59]. We would not expect it to be different for people living with HIV, including among those who profess to be indifferent to others’ dispositions. Indeed, evidence in other qualitative studies examining how people living with HIV manage and experience stigma demonstrate this cautious appraisal pattern [27,31].

These findings reveal that understanding how people orient to HIV stigma is useful not only for general counseling purposes, but also because of how a patient orients to HIV stigma might inform when and how clinicians choose to initiate HAART treatments. Given that non-adherence creates drug resistance and clinical failure, some care providers advocate delaying treatments until patients are “ready” to adhere [60,61]. Low concern for HIV stigma might be a sign of “readiness” [62], though it is worth noting that assessment of readiness has proven difficult [63], which is partly due to the failure to properly assess concerns for stigma. Research efforts have yielded several measures of HIV stigma, including the HIV/AIDS Stigma Scale [64], but these instruments are too cumbersome to administer during the brief course of a typical medical visit. As such, the development and testing of short assessments of stigma concern is needed.

Of course, the current study presents a key challenge in the proper assessment of Concern for HIV Stigma, as it reveals potential faults in how people self-report on this variable. Self-report data are notoriously inaccurate [65], especially when social desirability can influence a respondent’s answers [66,67]. It might be that competing identity concerns lead some, such as a number of participants in this study, to be less than honest regarding their true disposition. Further research is needed to assess the extent to which social desirability and other variables may be driving these inconsistent responses to concern for HIV stigma, especially if such assessments are to be used to determine when it is best to administer HIV medications.

Although this study benefited from a diverse sample of men and women across a variety of geographic and demographic backgrounds, these findings are limited in that they emerge from a qualitative research design and are therefore are neither generalizable nor comprehensive and cannot reveal, for instance, how stigma concerns may vary across demographic profiles. In addition, this study is limited in that it cannot longitudinally address how such concerns may change over the time people spend living with the disease. Further research looking at how concern for HIV stigma may vary across countries, demographic groups, and time frames will further inform our understanding of how to tackle these phenomena.

Finally, these findings encourage the reconsideration of a multitude of disclosure theories that are regularly employed in health behavior research. Specifically, these theories often emphasize the decision-making process and subsequent disclosure, privileging disclosure as the active component of the concealment-disclosure equation. Data presented here demonstrate that concealment is far from a passive, effortless state; rather, participants expressed a staggering array of demanding behaviors employed to conceal their HIV status. Taking medications in bathrooms, hiding medications in the trunks of cars, disguising medications, or throwing away medication instructions all illustrate how concealment can be highly intentional, effortful work. Adjusting our understanding of existing disclosure theories might refocus our attention on the plight that is faced by people managing stigmatized illnesses, as well as better inform the educational interventions and counseling employed to further help these people to survive.

## 5. Conclusions

Advances in HIV treatment have given hope to the possibility of a world without HIV. Researchers and clinicians must ally in the fight to understand and reduce the barriers to improper adherence in order to achieve this goal. The current study revealed the mechanisms of action by which stigma management can impair proper adherence, paving the way for subsequent research and possible counseling interventions that are designed to arm patients with understanding and tools for maximizing the effectiveness of their regimens. In addition, this study presents a case for re-examining the role of concealment in the extant disclosure literature, underscoring how concealment is no passive feat, but often the result of monumental effort. Taken together, these findings are presented as tools to use in the ongoing drive to eliminate the pernicious threat of HIV.

## Figures and Tables

**Table 1:** Demographics.

**Participants:**	93
**Sex:**	
Male	83
Female	10
**Race:**	
African American	48
White	32
Hispanic	12
Asian American	1
**Sexual Orientation:**	
Heterosexual	64
Bisexual	6
Homosexual	23
**Age:**	
Range	23–71
Mean	42.2
**Time Since Diagnosis:**	0–21 years
**Viral Load:**	
Known	77
Range	<50–1400
Average	412
Unknown	16
**AIDS Diagnosis:**	52

**Table 2:** Listing of Categorical Findings.

Category	Meaning
Orientation to Stigma	How people feel about HIV stigma
Perceived Prevalence of HIV Stigma	Belief in HIV stigma’s pervasiveness
Perceived Threat of HIV Stigma	Belief in HIV stigma’s severity
Concern for HIV Stigma	Degree of worry over HIV stigma
Stigma Management and Adherence	Coping that directly impairs adherence
Direct Impairment	Overtly missing or delaying medication
Medication Concealment–Public	Hiding medications in public
Medication Concealment–Private	Hiding medication in private
Medication Concealment–Storage	Inadvertently destroying medication
Indirect Impairment	Coping that indirectly impairs adherence
Social Support Management	Disrupting or facilitating support
Information Management	Hiding medication information
Information Avoidance	Foregoing sources of information

## Appendix A

1. What are situations that make it difficult to take your medications?
2. How might the stigma surrounding HIV affect the ways you or other people living with HIV take your medications?
3. Have you ever skipped a dose of your HIV medications so that other people won’t find out?
  - If so, how?
  - If so, why?
4. Are there any problems or drawbacks to other people helping you take your HIV medications?
5. How do you keep or manage information about your HIV medications?
